# Prediction of body fat increase from food addiction scale in school-aged children and adolescents: A longitudinal cross-lagged study

**DOI:** 10.3389/fpubh.2022.1056123

**Published:** 2023-01-06

**Authors:** Dan Wang, Hui Zhou, Yuzheng Hu, Yanfen Che, Xian Ye, Junqing Chen, Junfen Fu, Hongzhen Xu

**Affiliations:** ^1^The Children's Hospital, Zhejiang University School of Medicine, National Clinical Research Center for Child Health, Hangzhou, Zhejiang, China; ^2^Department of Psychology and Behavioral Sciences, Graduate School, Zhejiang University, Hangzhou, Zhejiang, China

**Keywords:** children, adolescents, food addiction, body composition, obesity, longitudinal study, cross-lagged analysis

## Abstract

**Objective:**

Food addiction (FA) is associated with a higher body mass index *z*-score (BMIZ) in children and adolescents; however, whether these two aspects evolve interdependently remains unknown. This study aimed to address this question using a cross-lagged study.

**Methods:**

Weight status, including BMIZ, fat content (FC), and visceral fat level (VFL), was determined in 880 children and adolescents (mean age = 14.02 years [range = 8.83–17.52 years]) at two-time points with an interval of 6 months. FA was characterized using the Chinese version of the dimensional Yale Food Addiction Scale for Children 2.0. Furthermore, FC and VFL were measured using direct segmental multi-frequency bioelectrical impedance analysis at each time point.

**Results:**

Higher FA was associated with increased BMIZ, FC, and VFL (*P* < 0.05). FA at T0 could predict increased FC at T1 (*P* < 0.05). The characteristics of females, primary students, and living in urban areas may aggravate the adverse effect of FA on weight status over time and age, particularly the increased VFL in participants aged > 14 years.

**Conclusion:**

Children and adolescents with a high FA level were at risk for weight gain attributed to increased FC, and the adverse effect could be aggravated with time and age. Novel FA-targeting interventions may help mitigate the risk of getting obesity.

Obesity is a revertible but easily relapsing condition because of several reasons, and food addiction (FA) emerged as a novel perspective for understanding eating behavior and was observed to correlate with weight status characterized by body mass index (BMI). However, how FA contributes to obesity remains unknown. In this longitudinal study, it was found that higher FA was associated with increased body mass index *z*-score (BMIZ), fat content (FC), and visceral fat level (VFL). The higher FA level at T0 could predicate increased FC at T1, and children and adolescents with a high FA level were at risk for weight gain, which was attributed to the increased FC. The characteristics of females, primary students, and living in urban areas may aggravate the adverse effect of FA on weight status over time and age. Moreover, the increase in VFL was more significant in participants 14 years in junior high school. FA could be integrated into the weight loss program in future studies to help children and adolescents lose weight, particularly to improve the loss of FC at an early age.

**What is already known about this subject?**

- Obesity is a revertible but easily relapsing condition because of several reasons, including psychological factors.
- Compulsive and overeating behavior results in failed weight loss programs, and the limited and transient effect of constraining food intake encourages more exploration of the underlying reasons.
- Food addiction (FA) has emerged as a novel perspective for understanding eating behavior and has been observed to correlate with weight status characterized by body mass index (BMI).

**What are the new findings in your manuscript?**

- Higher FA was associated with increased body mass index z-score (BMIZ), fat content (FC), and visceral fat level (VFL).
- The higher FA level at T0 could predicate increased FC at T1, and children and adolescents with a high FA level were at risk for weight gain, which was attributed to the increased FC.
- The characteristics of females, primary students, and living in urban areas may aggravate the adverse effect of FA on weight status over time and age, particularly in participants aged > 14 years.

**How might your results change the direction of research or the focus of clinical practice?**

- FA could be integrated into the weight loss program to help children and adolescents lose weight, particularly to improve the loss of FC.
- Special attention should be given to females and primary students living in urban areas because they are at high risk for weight gain attributed to increased fat. The adverse effect of FA, including the increased VFL, will be aggravated at the age of >14 years and over time.

## Introduction

The prevalence of overweight and obesity has become a public health problem globally, with a rapid increase in the number of children and adolescents (1). It was reported that, by 2016, approximately 124 million school-aged children and adolescents will be living with excessive weight status (2). In developing countries (2), such as China, the prevalence rate of excessive weight has increased from 14.5 to 35.3% during decades (3). The overweight and obesity onset in early childhood could lead to metabolic dysfunction (4) and chronic diseases in adulthood (5). Furthermore, it is associated with negative psychological development (6). Moreover, obesity could impair cognitive and social function and results in low self-esteem (7).

Obesity has been recognized as a revertible but easily relapsing condition (8), and the effect of energy restraint-based intervention was limited (9). Moreover, concerns about negative impacts on growth and other aspects of development owing to diet in children and adolescents have been raised (10), calling for alternative interventions. To explore potential long-term effects and safer interventions, a growing number of researchers have shifted to focus on the psychological causes of obesity (11, 12). Food addiction (FA) has become one of the emerging underlying psychological factors (13).

Individuals with FA were characterized by compulsive and impaired control of eating behavior (13). These symptoms could be more severe in school-aged children and adolescents owing to their impulsive traits (14). Additionally, several addictive behaviors started from an early age and lasted to adulthood (15), and longer addictive behavior duration would result in worse intervention outcomes. Therefore, considering FA in children and adolescents to prevent further consequences is significant.

Although several cross-sectional studies revealed a close correlation between FA and body mass index *z*-score (BMIZ) (16, 17), the potential causal relationship between FA and weight status has not been identified. Moreover, studies concerning FA were rare in children and adolescents, particularly longitudinal studies (18). Despite FA has remained controversial, increasing studies have shown that highly processed food could be as addictive as other substances, such as tobacco (19). With the changing food environment, particularly the easily accessible highly processed food, more empirical evidence concerning FA is needed (20).

Furthermore, BMIZ, which is calculated by weight and height, may be incomplete to characterize weight status (8). For example, the fat mass is typically underestimated using BMIZ (21), and the adverse consequences of obesity result from excessive fat mass deposited in the subcutaneous and viscera, but not simply body weight because other body compositions, including the muscle, also contribute to weight status but not obesity (22). Thus, to characterize weight status, particularly fat distribution, complementary measures, including body composition, should be included (22).

Therefore, this study aimed to explore the temporal longitudinal associations between FA and weight status, which are characterized using BMIZ and fat distribution, in school-aged children and adolescents and analyze the interactive effect of time and demographic characteristics in the associations.

## Methods

### Participants

Participants aged 8–18 years were enrolled in a primary school, junior school, and high school in a town in Eastern China. The following were the exclusion criteria: participants diagnosed with endocrine diseases, including hyperthyroidism, or another disease that may impact weight status (e.g., depression and anxiety); those who were receiving therapies, surgery, or other treatments in recent 3 months; those who were on a diet in recent 3 months; and those who did not participate owing to physical concerns, such as feet hurt.

### Measures

#### Demographic and screening information

Participants' demographic information, including age, gender, school, and inhabitation, were collected. A short screening form was used to exclude the participants who did not meet the criteria at each time of the survey.

#### Food addiction

The participants' additive-like eating behavior was characterized using the FA scores assessed by the Chinese version of the dimensional Yale Food Addiction Scale for Children 2.0 (dYFAS-C 2.0) (23). In 2018, Schiestl and Gearhardt (24) developed the dYFAS-C 2.0 based on the Diagnostic and Statistical Manual for Mental Disorders, fifth edition. The dYFAS-C 2.0 has 35 items rated using a 7-point Likert scale on each item (24). A higher score of dYFAS-C 2.0-C indicated a higher FA level (24). The Chinese version of dYFAS-C 2.0-C was translated and validated with good internal reliability of 0.934 (23). In this study, the average score was used to indicate the FA level. An FA score of >0.7 was categorized as a high risk of FA according to our previous study (23).

#### BMIZ and fat distribution

The BMIZ was calculated using WHO AnthroPlus, using the height and weight measured by the researcher, with BMIZ > 1 indicating overweight or obesity (25). The FC and VFL were assessed using a machine based on direct segmental multi-frequency bioelectrical impedance analysis (DSM-BIA). The machine was equipped with an eight-pedal electrode system and took direct impedance measurements based on the assumption that the human body is consisted of five interconnecting cylinders (26). A previous study showed that the internal consistency between DSM-BIA and the accurate dual-energy X-ray absorptiometry was >0.9 (27), indicating the machine's reliability in measuring body composition. The FC indicated the proportion of fat in the whole body weight; a higher FC implied that the fat contributed more to the weight and was influenced by age. However, several studies showed that the fat mass change was more dispersed with age compared with other body composition, such as bone mineral content and lean tissue mass, and was prone to be stable after the age of 10 (28, 29). Considering the role of growth and development of children and the wide range of age groups, FC > 18% and 28% for males and females were grouped as high FC based on the study of Fomon's et al. (30). The VFL indicated that fat accumulated among the abdominal viscera; a VFL of >10 indicated abdominal obesity according to the instructions of the BIA machine.

### Data collection procedure

A longitudinal study was conducted, and the participants' FA level and body composition were assessed twice at a 6-month interval. The participants' height, weight, and body composition were measured using the standard method after removing their coats, shoes, and socks (they must be barefooted to contact the plate electrode). To ensure accuracy, the height of the participants was measured twice, and the weight and body composition were measured using a body composition analyzer using the DSM-BIA method.

Following the measurements, each participant was provided with a card with individual information about their name, school, grade, and class, assigned number, measurements (height, weight, FC, and VFL), and a link to the online survey. They were allowed to complete the online survey at their most convenient time within 1 week.

Data collection was performed twice on May 2022 (T0) and November 2022 (T1) without any interventions being performed during the interval.

This study was approved by the ethics committee of the Children's Hospital of Zhejiang University (Approve Number: 2021-IRB-235). It was also approved by the schools and local education government. The participants and their families were informed about the survey through the school WeChat group (a social media platform). The introduction of the survey was recorded as a video, which depicted how the study aimed to explore the association between addictive eating behavior and weight status. Subsequently, the researcher measured the participants' height, weight, and body composition. An online survey must be completed by the participants within 1 week. Moreover, the video illustrated that the participants could quit at any time depending on their willingness. As compensation, to provide a reference of their growth status, the researcher calculated each participant's BMI and height percentages and provided their corresponding suggestion. Each participant could obtain a package of candy as a gift of participation. The participants' consent, both from the children and their parents or guardians, was acquired online before the online survey; only participants who agreed to participate in the survey and recognized their right to quit at any time could continue with the online survey.

### Statistical analysis

The participants' age, FA, BMIZ, FC, and VFL were expressed using means and standard deviations (SDs) or medians and interquartile ranges. The participants' gender, education level, inhabitation, and FA, BMIZ, FC, and VFL categories were summarized using numbers and proportions.

The longitudinal association of FA at T0 with BMIZ, FC, and VFL at T1 was explored using linear regression after adjusting for age, gender, education level, and inhabitation. To further explore and interpret the association between FA at T0 and BMIZ, FC, and VFL at T1, the variables were categorized into dichotomous ones. The longitudinal association between the FA category at T0 and BMIZ, FC, and VFL categories at T1 was explored using binary logistics regression, with covariate adjustment including age, gender, education level, and inhabitation.

The prospective associations between repeated measures of FA and BMIZ, FC, and VFL were analyzed using linear mixed models, and the interactive effects of the participants' demographic characteristics on the association between FA and weight status were also analyzed to explore the moderate effect of demographic characteristics between FA and weight status.

The temporal associations of FA and BMIZ, FC, and VFL were explored using structural equation modeling with an autoregressive cross-lagged design. First, the cross-lagged model tested the association of FA at T0 with BMIZ, FC, and VFL at T1, separately. Subsequently, the associations of BMIZ, FC, and VFL at T0 with FA at T1 were tested. These models were adjusted for the effect of covariates of age, gender, education level, and inhabitation. Standardized estimation was reported. *P* < 0.05 was considered statistically significant.

Statistical analyses were performed using SPSS (IBM Corp., Armonk, NY, USA) and Mplus version 7.1 (Muthén & Muthén, LA, CA, USA).

## Results

### Participant characteristics

**Figure 1:**
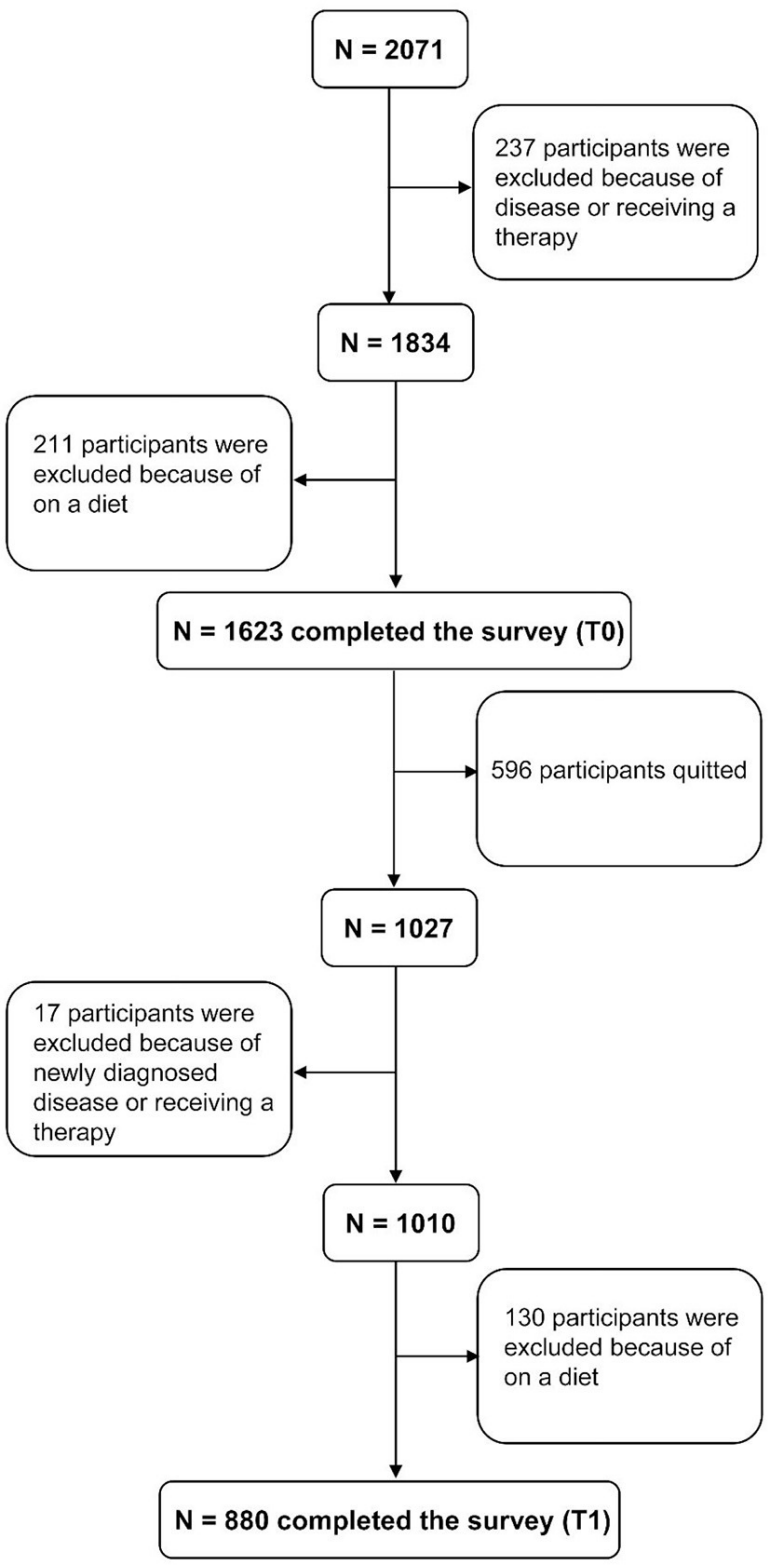
The flow chart of enrolment.

A total of 880 participants aged 8.83–17.52 years completed the two-time survey, with a mean age of 14.02 years (SD = 2.71) at baseline (the flow chart of enrolment and the characteristics of the participants who remained and dropped from the study are shown in Figure 1 and Table 1). A total of 450 (51.1%) and 430 (48.9%) male and female participants, respectively, were included, with 312 (35.5%), 128 (14.5%), and 440 (50%) primary school, junior high, and high school students, respectively. Half of the students (58.1%) inhabited the rural areas, and 41.9% inhabited the urban areas. The detailed demographic information and BMIZ, FC, VFL, and FA at T0 (May 2021) and T1 (November 2021) are presented in Table 2. In this study, the reliability of dYFAS-C 2.0-C was 0.953 at baseline.

**Table 1:** Comparison of the characteristics of retained and dropped participants.

**Variable**	**Group**			
	**Dropped participant** ***N* = 743**	**Completed T0–T1** ***N* = 880**	***t*/χ^2^/*Z*-value**	***P***
Age	13.27 (3.36)	14.02 (2.71)	4.861	< 0.001
Gender			14.084	< 0.001
Male	449 (60.4%)	450 (51.1%)		
Female	294 (39.6%)	430 (48.9%)		
BMIZ	0.41 (1.87)	0.12 (1.67)	4.572	< 0.001
FC	24.10 (14.80)	24.60 (13.98)	0.359	0.720
VFL	5 (5)	4 (4)	0.203	0.839
FA	0.49 (0.69)	0.40 (0.66)	2.401	0.016

Data at T0 are used to conduct analysis.

FA, food addiction; BMIZ, body mass index z-score; FC, fat content; VFL, visceral fat level.

**Table 2:** Characteristics and measurements of participants (*N* = 880).

**Demographic characteristics at baseline (T0)**	**Mean ±SD/median (IQR)/*n* (%)**
Age	14.02 ± 2.71 (8.83–17.52)
**Gender**	
Male	450 (51.1%)
Female	430 (48.9%)
**Education level**	
Primary school	312 (35.5%)
Junior high school	128 (14.5%)
High school	440 (50%)
**Inhabitation**	
Urban	369 (41.9%)
Rural	511 (58.1%)
**FA measurements**	
FA (T0)	0.40 (0.66 [0–3.06])
FA (T1)	0.43 (0.72 [0–3.89])
**Weight status indicators**	
BMIZ (T0)	0.12 (1.67 [−4.30 to 3.08])
BMIZ (T1)	0.44 (1.55 [−3.26 to 5.35])
FC (T0)	24.60 (13.98 [3.90–53.50])
FC (T1)	28.30 (13.27 [9.10–50.50])
VFL (T0)	4 (4 [1–20])
VFL (T1)	6 (5 [1–20])
**Variable categories**	
8–14 years	377 (42.8%)
>14 years	503 (57.2%)
High FA risk (T0)	257 (29.2%)
High FA risk (T1)	286 (32.5%)
Overweight/obesity (T0)	225 (25.6%)
Overweight/obesity (T1)	281 (31.9%)
High FC (T0)	479 (54.4%)
High FC (T1)	626 (71.1%)
Abdominal fat (T0)	85 (9.6%)
Abdominal fat (T1)	159 (18.0%)

FA, food addiction; BMIZ, body mass index z-score; FC, fat content; VFL, visceral fat level.

### Longitudinal association of FA at T0 with BMIZ, FC, and VFL at T1

In linear regression analysis, the FA at T0 was significantly associated with BMIZ (β = 0.600, *P* < 0.001), FC (β = 4.202, *P* < 0.001), and VFL (β = 2.248, *P* < 0.001) at T1, with covariates of baseline age, gender, education level, and inhabitation being controlled. Further generalized line model for repeated measures showed that the FA at T0 was significantly associated with the variance of weight status. The effect size of FA on BMIZ, FC, and VFL were 0.072, 0.070, and 0.089, respectively. The effect size of covariables was also evaluated, and the results were presented in Table 3.

**Table 3:** The longitudinal association between FA and weight status.

	**BMIZ**			**FC**			**VFL**		
	***F***	***P***	** $\eta_{p}^{2}$ **	***F***	***P***	** $\eta_{p}^{2}$ **	***F***	***P***	** $\eta_{p}^{2}$ **
Time	38.181	< 0.001	0.042	28.743	< 0.001	0.032	18.204	< 0.001	0.020
FA	68.041	< 0.001	0.072	66.077	< 0.001	0.070	84.775	< 0.001	0.089
Age	0.041	0.839	–	0.330	0.566	< 0.001	6.545	0.011	0.007
Gender	9.691	0.002	0.011	169.473	< 0.001	0.163	35.857	< 0.001	0.039
Education	11.218	< 0.001	0.025	10.339	< 0.001	0.023	4.088	0.017	0.009
Inhabitation	4.214	0.040	0.005	2.984	0.084	–	5.257	0.022	0.006

In this generalized line model, BMIZ, FC, and VFL (dependent variables) were repeated measures. The FA and age used in this analysis were T0 measures.

FA, food addiction; BMIZ, body mass index z-score; FC, fat content; VFL, visceral fat level.

In logistics analysis, the FA category at T0 was significantly associated with the BMIZ category (β = 1.120, odds ratio [OR] = 3.066, 95% confidence interval [CI]: 2.182–4.308, *P* < 0.001, classification accuracy: 73.6%), FC category (β = 0.627, OR = 1.873, 95% CI: 1.300–2.697, *P* = 0.001, classification accuracy: 72.3%), and VFL category (β = 1.136, OR = 3.115, 95% CI: 2.163–4.485, *P* < 0.001, classification accuracy: 82.0%) at T1, with covariates of age, gender, education level, and inhabitation being controlled.

### Progression effect of FA on BMIZ, FC, and VFL from T0 to T1 and interaction effect of participants' characteristics

In mixed linear model analyses, the variance of FA was significantly associated with the variances of BMIZ (*F* = 89.646, *P* < 0.001), FC (*F* = 88.620, *P* < 0.001), and VFL (*F* = 123.390, *P* < 0.001) from T0 to T1, with covariates of age, gender, education level, and inhabitation (Table 4).

**Table 4:** The main effect of associated variables of weight gain.

	**BMIZ**		**FC**		**VFL**	
	**F**	***P***	**F**	***P***	**F**	***P***
Time	24.376	< 0.001	53.509	< 0.001	37.979	< 0.001
FA	89.646	< 0.001	88.620	< 0.001	123.390	< 0.001
Age	0.016	0.900	0.472	0.492	13.308	< 0.001
Gender	17.379	< 0.001	311.818	< 0.001	66.818	< 0.001
Education	20.478	< 0.001	18.348	< 0.001	7.274	0.001
Inhabitation	4.842	0.008	3.945	0.020	7.189	0.001

In this mixed generalized model, FA (independent variable) and BMIZ, FC, and VFL (dependent variables) were repeated measures.

FA, food addiction; BMIZ, body mass index z-score; FC, fat content; VFL, visceral fat level.

**Figure 2:**
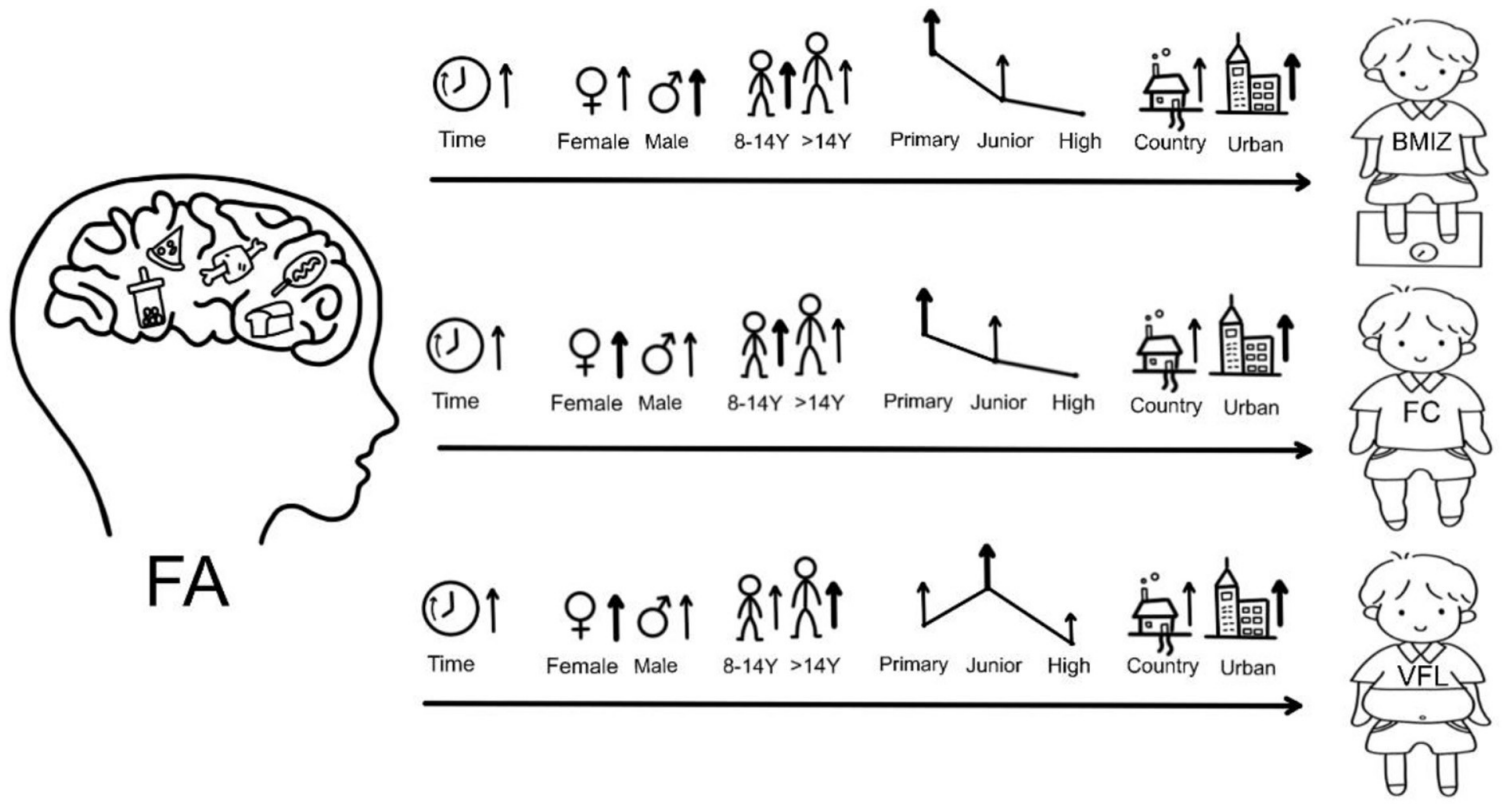
The moderate roles of participants' characteristics between FA and weight status (BMIZ, FC, and VFL). The “↑” indicates that this characteristic could aggravate the effect of FA on weight status characterized by BMIZ, FC, and VFL. The bold “↑” indicates more significant effect. FA, food addiction; BMIZ, body mass index z-score; FC, fat content; VFL, fat level.

Further analysis showed that time, age, gender, education level, and inhabitation could significantly moderate the effect of FA on BMIZ, FC, and VFL (*P* < 0.05) (Table 5). The participants aged 8–14 years experienced more increase in BMIZ and FC, whereas those aged > 14 years experienced more increase in VFL. Primary school students shared more increased BMIZ and FC, whereas junior high school students shared a more increased VFL, which was consistent with previous findings in 14-year-old participants. The participants living in urban areas were more likely to experience higher weight gain and fat distribution than those living in rural areas. The effect of FA on weight status indicated by BMIZ, FC, and VFL could be aggravated by time (Figure 2).

**Table 5:** The interaction effect between baseline FA and demographic characteristics on weight.

	**BMIZ**		**FC**		**VFL**	
	**β**	***P***	**β**	***P***	**β**	***P***
**Time point**						
T0	0.371	< 0.001	2.354	< 0.001	1.661	< 0.001
T1	0.507	< 0.001	4.500	< 0.001	2.756	< 0.001
T0 vs. T1	0.136	< 0.001	2.146	< 0.001	1.095	< 0.001
**Age**						
8–14	0.841	< 0.001	4.694	< 0.001	1.478	< 0.001
>14	0.333	< 0.001	3.246	< 0.001	2.414	< 0.001
8–14 vs. >14	0.508	< 0.001	1.448	< 0.001	0.936	< 0.001
**Gender**						
Male	0.540	< 0.001	0.113	0.800	1.425	< 0.001
Female	0.349	< 0.001	7.334	< 0.001	3.019	< 0.001
Female vs. male	0.191	< 0.001	7.221	< 0.001	1.594	< 0.001
**Education level**						
Primary school	1.165	< 0.001	6.200	< 0.001	1.778	< 0.001
Junior high	0.379	< 0.001	3.438	< 0.001	2.488	< 0.001
High school	0.014	0.899	1.192	0.155	0.982	0.004
Primary vs. junior vs. high school	0.786	< 0.001	2.762	< 0.001	1.506*	< 0.001
**Family inhabitation**						
Urban	0.933	< 0.001	5.705	< 0.001	2.601	< 0.001
Rural	0.283	< 0.001	2.847	< 0.001	2.042	< 0.001
Urban vs. rural	0.650	< 0.001	2.858	< 0.001	0.559	< 0.001

In this analysis, the independent variable is food addiction.

A generalized model for repeated measures is used to estimate the interaction effect between FA and demographic information on BMIZ, FC, and VFL. ^*^The interaction effects among education groups across time are significantly different from each other after adjusting for pairwise comparisons.

BMIZ, body mass index z-score; FC, fat content; VFL, visceral fat level.

### Cross-lagged temporal relationship between FA and BMIZ, FC, and VFL

**Figure 3:**
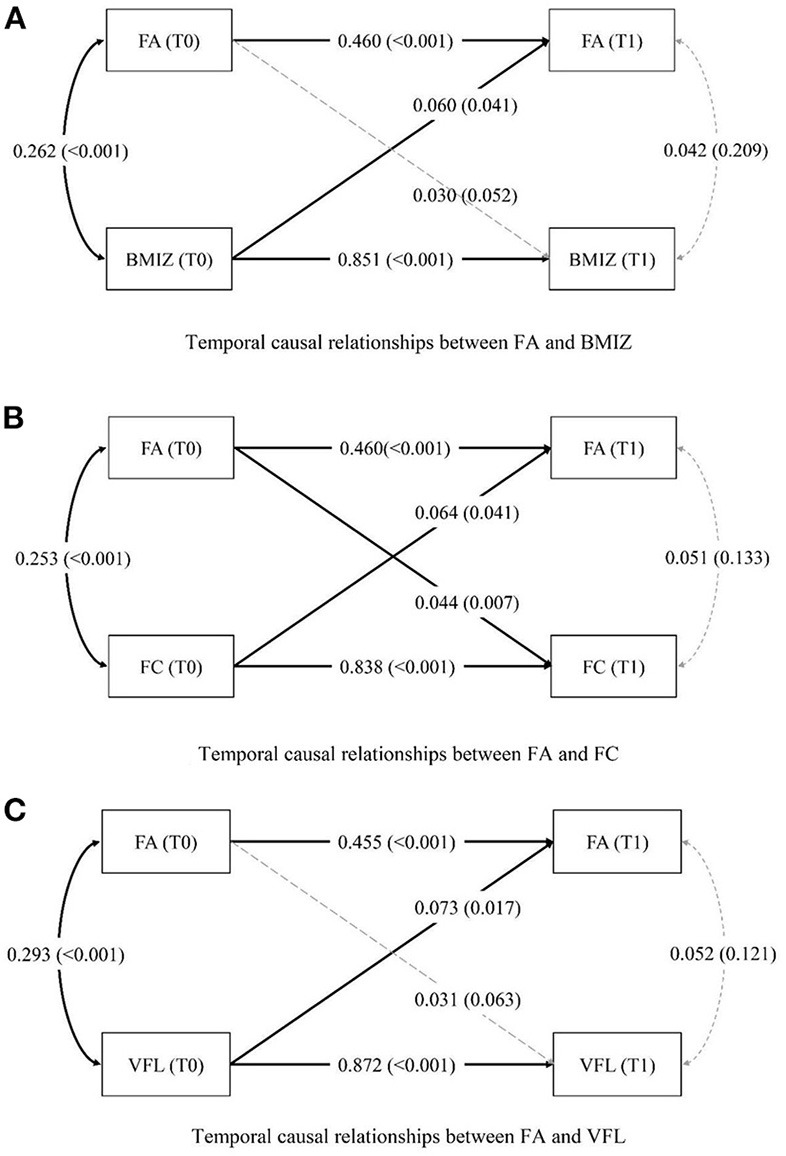
**(A–C)** Temporal and causal relationships between FA and weight status. The *P*-values are presented in brackets. The concert line indicates a significant pathway, whereas the dashed line indicates a nonsignificant pathway. FA, food addiction; BMIZ, body mass index *z*-score; FC, fat content; VFL, visceral fat level.

FA at T0 was directly associated with FC at T1 (β = 0.044, *P* = 0.007), see Figure 3B. BMIZ (β = 0.060, *P* = 0.041), FC (β = 0.064, *P* = 0.041), and VFL (β = 0.073, *P* = 0.017) at T0 were directly associated with FA at T1, with covariates of age, gender, education level, and inhabitation being controlled (Figures 3A–C). The estimation of autoregressive and cross-lagged variables is presented in Table 6. The autoregression effects of FA, BMIZ, FC, and VFL at T0 and T1 were significant (*P* < 0.001). The cross-lagged effect was significant between FA and FC (*P* < 0.05).

**Table 6:** Autoregressive cross-lagged temporal analyses of FA with BMIZ, FC, and VFL at T0 and T1.

	**Standard estimate**	**Standard error**	***t***	***P***
**Autoregressive**				
FA (T0) → FA (T1)	0.460	0.027	17.356	< 0.001
BMIZ (T0) → BMIZ (T1)	0.851	0.010	81.649	< 0.001
FC (T0) → FC (T1)	0.838	0.012	68.604	< 0.001
VFL (T0) → VFL (T1)	0.872	0.011	81.337	< 0.001
**Cross-lagged**				
FA (T0) → BMI (T1)	0.030	0.016	1.940	0.052
BMI (T0) → FA (T1)	0.060	0.029	2.041	0.041
FA (T0) → FC (T1)	0.044	0.017	2.675	0.007
FC (T0) → FA (T1)	0.064	0.031	2.039	0.041
FA (T0) → VFL (T1)	0.031	0.017	1.858	0.063
VFL (T0) → FA (T1)	0.073	0.031	2.385	0.017

FA, food addiction; BMIZ, body mass index z-score; FC, fat content; VFL, visceral fat level.

## Discussion

This study explored the temporal longitudinal associations between FA and weight status characterized using BMIZ, FC, and VFL. The results showed that children and adolescents with a high risk of FA were 2–3 times (OR: 1.873–3.115) more likely to be diagnosed with excessive weight status than those with low FA risk. FA was a distinguishable feature of participants with excessive weight, and higher FA was significantly associated with increased FC. The characteristics of females, primary school students, different age stages, and living in urban areas could exacerbate the effect of FA on weight gain and fat distribution.

Previous studies showed that FA is prevalent in children with overweight or obesity (31, 32). It was reported that approximately 19% of school-aged children with overweight/obesity were categorized with FA, even in the general population; however, 12% of children were found to have FA, and the overall prevalence of FA was 13–18% (18, 31). In our previous study, we identified that an average score of 0.7 was strongly associated with higher BMIZ and could be an indicator of high FA risk (23). In this study, participants with a high risk of FA at baseline had a 2–3 times probability of being diagnosed as excessive weight during follow-up. Further analysis of repeated measures showed that the higher level of FA was associated with increased BMIZ, FC, and VFL and resulted in weight gain and fat distribution. These results showed that FA was closely associated with increased weight and may play a role in maintaining a higher level of weight. The results were consistent with Almeida's weight loss intervention study, with the conclusion that FA contributed to the maintenance of weight and difficulties in weight loss (33).

Moreover, the effect of FA on weight status variance was moderated by time, participants' age, gender, education level, and inhabitation. After 6 months, the results showed that the adverse effect of FA on weight status may be more obvious than before. This indicates that with prolonged time, the FA may contribute to more rapid weight gain and fat distribution. Thus, timely intervention of FA is significant in a weight control program. This result was consistent with those of other studies on addiction, which implied that a longer duration of addiction symptoms would result in several adverse consequences (34), in the case of FA, leading to an aggressive weight gain, particularly the increase in fat mass.

This study showed that the effect of FA on weight status could be moderated by different age stages. Compared with participants aged > 14 years, the participants aged 8–14 years were more likely to experience an increased BMIZ and fat distribution with higher FA levels; however, those aged > 14 years were prone to experience more VFL increase due to severe FA over time compared with younger age group. The different effects could be attributed to the different body composition development features across ages (35). The subcutaneous fat rapidly developed before the age of 12, particularly before 10 years old (28, 29), and tended to persist at its peak at 13–14 years without a significant variance in a wide percentile scope (35). Conversely, the visceral adipose tissue still tended to increase after 14 years, particularly in individuals with visceral fat tissue at the 50 percentile and above (35), causing a more adverse effect on metabolic function (36). Moreover, visceral fat was significantly associated with chronic disease and cancer compared with subcutaneous fat (37), suggesting the significance of paying attention to the VFL at a young age in children with high FA risk. Additionally, particularly at the age of 14, a more detailed physical check should be performed to detect the adverse effect of FA and facilitate timely intervention.

In this study, although male participants' BMIZ was affected more by the FA, the female participants were more likely to experience an increased fat distribution rather than weight gain than the male participants, which was consistent with the results of previous studies (38). This may be because of the development feature of females (39) and gender-specific fat distribution patterns (40). Owing to the appearance of secondary sexual characteristics, females were prone to deposit adipose tissues during development (41). This may make the female participants easily impacted by the FA. Moreover, a study on the adult population showed that female participants were more prone to FA than their male counterparts (42). Gender itself may play a part in the efficiency of FA on weight status.

In this study, the primary students were affected more by the effect of FA than the junior and high school students, indicating that the impact of FA could be moderated by the education level. On one hand, the inhibitory ability will be improved with the development of brain function as the education level was improved (43). On the other hand, the intensive image attention emerging from junior high school primarily would help the students to shift their interest from food to other social activities (44), which may help them to decrease the effect of FA.

Moreover, students living in rural areas were less affected by the FA than their counterparts living in urban areas. This may be because of the limited number of shops or supermarkets selling highly processed snacks in rural areas. This indicated that the social environment was also significant for alleviating the effects of FA, and constraining the sales of highly processed food without adequate nutrition, such as sweetened drinks and chips, may be helpful (45). A multicenter intervention conducted in China also showed the efficiency of constraining certain food sales in primary schools (46).

After controlling the abovementioned covariates, the bidirectional association between FA and FC was clear in this study. Furthermore, the direct association between BMIZ and VFL to FA was significant. The results indicated that the FA was a distinguishable feature of participants with higher BMIZ and fat distribution. Additionally, the bidirectional association between FA–BMIZ and FA–VFL was close to a significant value, suggesting the potential of FA for the predicated temporal variance of both BMIZ and fat distribution. The only bidirectional association between FA and FC may be because of the sensitive change in FC compared with BMIZ and VFL. The prediction effect was similar to the effect of behavior problems on BMIZ in preschoolers (0.044 vs. 0.09) (47). The FC and VFL at baseline were both directly associated with FA and may also show the dysregulation of hormone and brain function resulting from increased adipose tissue. Studies showed that the increased fat mass could enhance insulin resistance (48), thereby leading to elevated energy intake. Moreover, a study showed that decreased glutamate/creatine ratio was observed in the hypothalamus of adolescents with obesity, indicating appetite regulation dysfunction (49). This may promote eating behaviors in children and adolescents with overweight and obesity and contribute to severe FA. Therefore, in a weight loss program, recording the variance of adipose distribution may be more effective than focusing on weight only. This could not only help the participants to lose weight but also decrease the effect of FA and consolidate the effect of weight loss in the long run.

## Limitations

This study had several limitations. First, although the results from a longitudinal design were more robust than those of a cross-sectional one, the causal relationship could not be firmly identified; only the temporal association of FA and weight status could be identified in this study. Second, the fat distribution was measured based on the bioelectrical impedance analysis method rather than dual X-ray, which may reduce the accuracy of the measurement. However, given the safety (50) and interchangeability between the two methods in a population-based study (51), the results were credible. Third, other covariates, including food intake and physical activities, were unavailable in the participants owing to large missing data; further studies should evaluate these important covariates as required from to ensure the integrity of the data set, and the follow-up duration may not be long enough to detect more variances. Lastly, the effect of pubertal development was not evaluated in this study; however, given the relatively short follow-up period and that most participants were above the age of 10 (92.8%), the results could be less influenced by the factor of pubertal development.

## Conclusion

FA was predictive of FC increase and associated with increased BMIZ and VFL, and the characteristics of being female, primary school students, and those living in urban areas were factors that could aggravate the effect of FA on weight gain and fat distribution. The FA was significantly associated with BMIZ, FC, and VFL across different age groups. However, the different effects of FA on weight status between age groups should be noticed. The intervention of FA should be incorporated into weight loss programs to help children and adolescents with overweight or obesity lose weight and ensure a prolonged effect.

## Data availability statement

The raw data supporting the conclusions of this article will be made available upon reasonable request.

## Ethics statement

The studies involving human participants were reviewed and approved by the Children's Hospital of Zhejiang University. Written informed consent to participate in this study was provided by the participants' legal guardian/next of kin.

## Author contributions

DW research design, data collection, data analysis, and draft. HZ and YH revision. YC data visualizaztion. XY and JC data collection. JF resource and supervision. HX supervision. All authors contributed to the article and approved the submitted version.
